# 

**Published:** 1880-04-20

**Authors:** 


### EDITORIAL AND MISCELLANEOUS.

☞*NOTICE.*—Thanks to all who have remitted their subscriptions. There are many who have not done so. Our friends will please consider that the present improved condition of business affairs, while it makes it easier for them to pay, is hard upon publishers, by reason of the large advance on paper and printing material, while our terms remain the same. Be prompt, therefore, friends, and send up your little dues to the

MANAGING EDITOR.

---

☞The pressure, just at this time, for choice pages by advertisers compels the insertion of one or two advertisements amid our reading matter, but they are so inserted as to be readily omitted in binding the volume, and we trust, therefore, will cause no complaint.

---

*The Georgia Medical Association* meets in Augusta on the 21st inst., too late for the proceedings to appear in the present number of our Journal. We trust that the meeting will be a large and interesting one. As to the reception of the delegates there need be no apprehension, as our Augusta brethren are renowned for kindness and the most generous hospitality. Unavoidable circumstances will prevent the attendance of the Editors of the Record at the Association, which they greatly regret.

---

*The Literary Success of the Century.*—An eminent English authority recently pronounced *Scribner's monthly* "the greatest literary success of the century." The New England *Journal of Education* says . "America may well be proud of such a magazine." The *Illustrated London News* considers it "one of the marvels of the day." The *London Illustrated* (penny) *Paper* says : "With its inimitably finished gems of drawing and engravings, it is the wonder and admiration of the art-world.

The subscription price is $4.00 a year.

---

*Compliments to Dr. H. B. Lee.*—At a social repast recently given by Dr. Thos. F. Houston, in honor of Dr. H. B. Lee, a number of medical gentlemen being present, the following resolution, offered by Dr. Thos. S. Powell, was unanimously adopted.

Whereas our fellow citizen and medical friend, Dr. H. B. Lee, Auxiliary Prof. of Obstetrics and Diseases of Women in the Southern Medical College, has decided to remove from our midst to a distant city ;

*Resolved*, That we regret the move and will miss him in our social and medical circles. And that we cordially commend him to the Profession and to the community wherein his lot may be cast, as a gentleman of intelligence and honor, worthy of the confidence of medical brethren, and of patronage and success in the profession, or in whatever vocation he may choose to engage.

---

*The Erythroxylon Coca.*—We invite attention to the advertisement of this article in the present number of the Journal, by the Liebig Laboratory and Chemical Works Company. The New York Medical Journal thus speaks of this article :

"The medical profession is naturally, and very properly so, conservative in its acceptance of new theories, and especially so when extravagant claims are made in behalf of unknown remedies. Especially reluctant have many been with reference to the Coca. The powers claimed for it have seemed quite incredible, and no doubt it would have been dismissed without so much as a second thought, had not such names as Humboldt, Christison, and other equally eminent scientists, travelers and physicians, lent their names to it. The Liebig Laboratory and Chemical Works Company now offer it to the profession in a form which presents many advantages. Thus it is, for instance, well understood that the active principle of the leaf is extremely volatile, and that it is, in consequence, quite or wholly worthless when it reaches us. The Liebig Company overcome this by using in the tonic named only the fluid extract, prepared directly from the freshly-picked leaf. The beef contained in the tonic is from carefully-selected healthy bullocks, and the Company claim that it contains a much larger per centum of albuminoid and nutritive elements than is to be found in other beef tonics and extracts. The coca and beef are dissolved in a choice quality of sherry wine. Whether or not Coca Beef Tonic is all that the Company claims, there can be no doubt that the endorsements of numerous medical men of prominence, who have used it, which the Company display in their offices, indicate that it has merits. The formula is on the bottles."

---

*American Medical Association.*—At a meeting of the Committee of Arrangements of the American Medical Association, held in New York December 3d, 1879, the following Committees were appointed :

*Commitee on Building.*—Drs. M. A. Pallen. M. H. Burton, W. M. Polk.
*Reception Committee.*—Drs. Jas. C. Hutchinson. W. M. Polk, M. A. Pallen.
*Committee on Finance.*—Drs. Stephen Smith, A. A. Smith, W. R. Gillette, R. F. Weir, M. H. Burton, E. H. Parker.
*Committee on Business.*—Drs. M. A. Pallen, Jas. C. Hutchinson, Stephen Smith.
*Committee on Invitations.*—Drs. W. M. Polk, R. F. Weir, E. H. Parker.
*Committee on Entertainment.*—Drs.C.J. Pardee, M.A. Pallen, R.F.Weir.
*Committee on Printing.*—Drs. R.F.Weir, Stephen Smith, W.R. Gillette.

S. O. VANDERPOEL, M.D., *Chair. Com. Arrangements.*

W. R. GILLETTE, M.D., *Sect'y Com. Arrangements.*

### *RECEIPTED.*

[Receipts not acknowledged privately are entered here.]

1880..—Drs. J. S. Milling; John Hardeman; C. W. Keller; R. L. Seals; S. G. Dilburn; R. D. Caldwell; J. D. Groover; M. V. B. Miller; J. T. McDowell; J. S. Stidham; A. A. Lyon; H. Allison; J. M. Walker; W. S. Morgan; J. S. Bacon; J. M. Pinkston; A. J. Kolb; I. H. Gunter: A. A. Hill; L. M. Lovelace; E. H. M. Parham; W. R. Brawner; B. R. Doyle; A. R. Oglesby; R. T. Walker; L. M. Coleman; J. T. McDowell; Wm. Law; B. F. Phepps.

